# Identification, characteristics and rice growth promotion of a highly efficient cellulolytic bacterial strain, *Cellulomonas iranensis* ZJW-6, isolated from paddy soil in central China

**DOI:** 10.3389/fmicb.2023.1152966

**Published:** 2023-03-22

**Authors:** Lei Wu, Songhao Che, Xueting Qin, Yufeng Xu, Shiqi Tian, Yuan Zhu, Jian Song, Yunpeng Guan, Dongchao Wang, Meikang Wu, Xue Yang, Zhihai Wu, Meiying Yang

**Affiliations:** ^1^College of Life Science, Jilin Agricultural University, Changchun, Jilin, China; ^2^Faculty of Agronomy, Jilin Agricultural University, Changchun, Jilin, China

**Keywords:** ZJW-6, *Cellulomonas iranensis*, lignocellulose degradation, improving the soil, promoting the growth of rice

## Abstract

The microbial degradation of lignocellulose is the best way to treat straw, which has a broad application prospect. It is consistent with the idea of agricultural sustainable development and has an important impact on the utilization of biomass resources. To explore and utilize the microbial resources of lignocellulose degradation, 27 lignocellulose degrading strains were screened from 13 regions in China. ZJW-6 was selected because of its 49.6% lignocellulose weight loss rate. According to the theoretical analysis of the experimental results, the following straw degradation conditions were obtained by ZJW-6: nitrogen source input of 8.45 g/L, a pH of 8.57, and a temperature of 31.63°C, the maximum weight loss rate of rice straw could reach 54.8%. It was concluded that ZJW-6 belonged to *Cellulomonas iranensis* according to 16S rRNA-encoding gene sequence comparison and identification. ZJW-6 is a Gram-positive bacterium that grows slowly and has a small yellowish green colony. To explain the degradation mechanism of lignocellulose, the experiment of enzymatic properties of the strain was prepared and carried out. It was discovered that ZJW-6 has an excellent ability to degrade cellulose, hemicellulose, and lignin, with cellulose and hemicellulose loss rates reaching almost 50% in 4 days and lignin loss rates reaching nearly 30%. Furthermore, ZJW-6 demonstrated lignocellulose degradation under aerobic and anaerobic conditions, indicating the strain’s broad application potential. ZJW-6 was found to be more effective than ordinary humic acid in improving rice soil (available phosphorus, available nitrogen, organic matter) and promoting rice growth in a rice pot experiment (increasing root-shoot ratio, root activity, chlorophyll content and net photosynthetic rate). ZJW-6 plays an important role in promoting the development and utilization of straw resources. It has important significance for the advancement of green agriculture.

## 1. Introduction

The increasing use of bioenergy in industry has renewed interest in cellulose degrading bacteria. To date, lignocellulosic biomass, such as agricultural leftovers of rice straw, which is naturally rich in cellulose, is considered as the preferred choice for bioproduction due to its low cost and environmental friendliness (Naofumi Kamimura et al., 2019; Prasad et al., 2019; Siracusa, 2019; Kashif et al., 2020; Yingqun Ma and Liu, 2020). However, over the past decades, there has been an unavoidable reliance on expensive commercial pure enzymes for efficient conversion of cellulosic materials because only a limited number of microorganisms ranging from Archaea to Eukaryotes have been determined to be capable of cellulose biodegradation, despite the biomass’s ubiquitous presence in the biosphere, which posed a major barrier to its bioconversion (Congfeng Xu et al., 2019). Thereby, characterization of wild-type isolates from natural niche is required to improve our understanding, allowing for the improvement of existing efficiency in commercial cellulose degradation while also protecting the environment. Furthermore, the underlying mechanisms of microbial cellulose degradation remain unknown.

*Cellulomonas* species, unique cellulose degraders, Gram-positive bacteria’s with high GC content, were not commercially significant until recently, as notions of biotransformation of cellulosic agricultural wastes arose (Dahal et al., 2022; Gui Zhang et al., 2022; Siriatcharanon et al., 2022). *Cellulomonas fimi*, in particular, is one of the few facultative anaerobes identified to date that is capable of degrading cellulose at oxygen levels as low as 1%, which was thought to be more favorable than aerobic cellulose-degrading fungi in this regard (Christopherson et al., 2013).

Three kinds of enzymes synergistically interplay exerting cellulase activities: the primary enzyme, endoglucanase was responsible for cleaving the β-1,4-linkage of the cellulose polymer; Exoglucanase was responsible for the release of cellobiose from the nonreducing end of the cellulose; and β-glucosidase acted to completely degrade oligosaccharide and cellobiose into glucose (Kleine and Bolm, 2013; Kaur et al., 2017; Sinha et al., 2019). There are a few examples of how cellulases from *Cellulomonas* species work. Rajoka and Malik discovered that the cellulases of *C. fimi* had a two-domain structure that performed both catalytic and binding functions to break down the 1,4-beta-glycosidic-linkages of cellulose (Rajoka and Malik, 1997). A review by Himmel et al. indicated that aerobes and anaerobes adopted distinct pathways for cellulose degradation (Himmel et al., 2017). Aerobes, such as Actinobacteria, would excrete the enzyme into the extracellular environment, whereas anaerobes, with cellulosomes bound on the surface as a platform for clenching catalytic units, required close contact with the cellulose substrates. Christopherson et al. studied the genomics of three type strains of *Cellulomonas* species, *C. fimi* ATCC 484^T^, *C. gilvus* ATCC 13127^T^, and *C. flavigena* ATCC 482^T^ and suggested that these *Cellulomonas* spp. secreted extracellular cellulases aerobically and anaerobically, without interacting with cellulosomic structures on the surface of the latter, which contradicted the earlier version proposed for anaerobic cellulose decomposition (Christopherson et al., 2013).

In this study, a wild-type, highly efficient rice straw-degrading strain, *Cellulomonas iranensis* ZJW-6, isolated from a rice field in Zhangjiawan, Shanxi Province, China. Through the study of its cellulase activities under aerobic and anaerobic conditions, the characteristic cellulolytic machinery of *Cellulomonas* genus was further elucidated. Meanwhile, the rice pot experiment revealed that strain ZJW-6 could promote rice growth under the condition of straw returning to the field, effectively improving not only the soil condition, but also the root-shoot ratio, chlorophyll content, net photosynthetic rate, and root activity.

## 2. Materials and methods

### 2.1. Materials for experiments

Soil samples were collected from 13 sites across the central and northern China (details of the locations can be found in Supplementary Table S1). Soil samples were collected with a tube sampler at each location, transported to the laboratory in black plastic bags, and stored in the dark at 4°C. Rice straw was collected from Jilin Agricultural University’s agricultural experimental field. The straw was washed in the laboratory, dried at 60°C, and then chopped into 5 cm shards for the weight loss experiment.

### 2.2. Isolation and screening of cellulose-degrading strains

#### 2.2.1. Isolation of strains

According to Zhang’s method (Zhang Hai-Yan and Meng, 2020), strain isolation was performed. Prior to isolation, the soil sample was air-dried at room temperature for 14 days, ground into powder, and suspended in sterile distilled water, followed by a standard serial dilution in water. 10 g soil samples were initially preincubated with 25 g sterile glass beads in a conical flask containing 90 ml sterile water at 30°C, 160 rpm for 24 h, followed by standing still for 1 h, then the supernatant was collected and diluted with sterile water at 10^−3^, 10^−4^, and 10^−5^, respectively, then spread on 15 ml the primary screening medium (CMC 15 g/L, K_2_HPO_4_ 1 g/L, NH_4_NO_3_ 1 g/L, MgSO_4_ • 7H_2_O 0.5 g/L, Peptone 0.5 g/L, Agar 20 g/L, pH = 7) for lignocellulose degrading. Plates were incubated at 30°C for 48 h aerobically, well separated fully grown single colonies were picked up and sub-cultured by streaking on the lignocellulose degrading medium (Rice straw 10 g/L, K_2_HPO_4_ 2 g/L, NH_4_NO_3_ 4 g/L, MgSO_4_ • 7H_2_O 0.5 g/L, Peptone 10 g/L, beef extract 5 g/L), grown at 30°C for 72 h. The strain can be retained after numerous generations of culture until it is stable.

#### 2.2.2. Screening and identification of strain ZJW-6

##### 2.2.2.1. Screening by straw degradation ability

Rice straw degradation was carried out as follows: strains were cultured in liquid medium containing rice straw for lignocellulose degradation. The weights of the straw before and after microbial treatment were recorded. The bacteria-straw mixture was cultured at 30°C for 4 days, at 160 RPM. At the end of the incubation, the treated straw was filtered and washed before being dried in an oven at 60°C until its weight remained constant (Wenjie Gu et al., 2012).

The experiments were carried out in three replicates for each isolated bacterial strain, and the weight loss rate was estimated using the following formula:

Lignocellulose weight loss rate (%) = (original weight of straw − weight of straw after degradation)/original weight of straw × 100%.

##### 2.2.2.2. Optimization of straw degradation conditions by ZJW-6

To optimize the straw degradation rates of strain ZJW-6, three parameters were chosen: temperature (A), pH (B), and nitrogen source addition amount (C). The weight loss rate of rice straw was used as the index. The effects of nitrogen source addition account (6 g/L, 8 g/L, 10 g/L, 12 g/L, 14 g/L), pH (6, 7, 8, 9, 10), and temperature (25°C, 28°C, 31°C, 34°C, 37°C) on rice straw degradation rate were investigated. Each experimental group’s straw weight loss rate was measured when the inoculation level was 1% and the liquid medium for lignocellulose degradation was 30°C at 180 RPM for 4 days. Under each condition, three replicate samples were prepared, and the average weight loss rate was estimated using the same approach as in 2.2.1.

Based on the experimental results, temperature (A), pH (B), and nitrogen source addition account (C) were chosen as independent variables, straw weight loss rate was chosen as response value, and Box–Behnken experimental design was used in design expert 8.0 program (Stat-Ease, Inc., Minneapolis, MN, United States). The best degradation conditions were obtained based on the results of the response surface analysis.

### 2.3. Identification of the *Cellulomonas* strain, ZJW-6

The strain with the highest efficiency in straw degradation was developed and identified using Kim’s 16S rRNA-encoding gene sequencing technique (Kim et al., 2000). Specifically, chromosomal DNA from the strain was isolated using the CTAB / NaCl technique and utilized as a template for 16S rRNA-encoding gene amplification. PCR reaction conditions: 4 min at 94°C; 30 s at 94°C, 30 s at 53°C and 30 s at 72°C for 30 cycles; 10 min at 72°C. The 1,424 bps amplification products were validated by Sanger sequencing (Sangon Biotech corporation, Shanghai, China). Phylogenetic analysis was conducted on the obtained 16S rRNA-encoding gene sequence of ZJW-6 aligned with those of other *Cellulomonas* species in the NCBI database 16S rRNA-encoding gene using bioedit software (7.0.5.3, Borland) and the maximum-likelihood approach within MEGA 7.0 (Kumar and Khanna, 2015).

### 2.4. The characteristics of ZJW-6

#### 2.4.1. Degradation of cellulose, hemicellulose and lignin of straw by strain ZJW-6

The concentrations of cellulose, hemicellulose and lignin in rice straw were determined independently using the Van Soest method (van Soest and McQueen, 1973; van Soest et al., 1991), as described below:

1. After washing and filtering, the straw residues were dried in an oven at 60°C to achieve constant weight (W1)
2. The dried residual straw was weighed and placed in a 100 ml iodine flask containing 50 ml neutral detergent, then boiled in a pressure cooker for 1 h at 100°C. Following that, the samples were transferred into the funnel for filtration, and the filtered residue was washed with hot water over 90°C until the neutral pH was around 7.0. To ensure that no neutral detergent remained, the sample was rinsed with acetone 2–3 times before being returned to the oven and dried at 60°C to a constant weight (W2)
3. The straw sample from (2) was put in a 100 ml iodine flask with 50 ml of 2 mol/l HCl, and then boiled in the pressure cooker at 100°C for 50 min, then poured into a funnel and filtered, then the filter residue was washed until it became neutral, followed by washing with acetone 2–3 times, then the samples were placed in the oven and dried at 60°C to a constant weight (W3)
4. The dried straw samples from (3) were put in a beaker with 5 ml of 72% sulfuric acid that had been stored in the refrigerator ahead of time, hydrolyzed at room temperature for 3 h, then 50 ml distilled water was added and left to stand for 20 h. The weighed sand core crucible (W0) was then used for filtration and rinsed with distilled water until the pH reached neutral (7.0). The sand core crucible containing filter residue was the placed in an oven and dried to a constant weight at 60°C (W4)
5. The dried straw residue from (4) was combined with the sand core crucible in a muffle furnace, ached at 550°C for 4 h, and then added to the dryer to cool to a consistent weight (W0 + W5)

The cellulose, hemicellulose, and lignin contents in the samples were estimated using the formula:

Cellulose content=(W2−W3)/W1×100%

Hemicellulose content=(W3−W4−W5)/W1×100%

Lignin content=W5/W1×100%

The weight loss rates of three substances were then estimated based on their content in different samples.

#### 2.4.2. Growth curves of ZJW-6 under aerobic and anaerobic conditions

Strain ZJW-6 was cultured in 200 ml of LB culture medium until the OD_600_ reached 0.6–0.8. The bacterium liquid was then inoculated into lignocellulose degradation liquid medium at 1% inoculum level for shaking table culture, and cultured for 72 h at 30°C and 160 rpm under aerobic and anaerobic conditions, respectively. Anaerobic culturing was performed in anaerobic bags (C-35, Mitsubishi Corporation, Japan). The bacteria solution’s light absorption value (OD) at 600 nm was determined using the cellulose degradation liquid medium as a blank control, and each sample was repeated three times. The straw in the culture medium was removed by 200 mesh filter screen before the light absorption value of the bacterial solution was measured. Every 12 h, the light absorption value (OD) was measured. The optical absorption value of each sample should be between 0 and 1. If the result is outside of this range, the sample must be diluted, and the final absorbance value is calculated by multiplying the absorption value of the diluted sample by the dilution multiple. Light absorption value and culture time was used to determine the growth curve.

#### 2.4.3. Cellulase characteristics of ZJW-6 under aerobic and anaerobic conditions

##### 2.4.3.1. Extraction of enzyme solution

Strain ZJW-6 was cultured in 200 ml of LB culture medium until the OD_600_ reached 0.6–0.8. The bacteria were centrifuged and resuspended in a straw liquid fermentation medium. The suspension was evenly divided between two 100 ml straw liquid fermentation media and cultured for 96 h under aerobic and anaerobic conditions. Anaerobic culturing was performed in anaerobic bags (C-35, Mitsubishi Corporation, Japan). For 5 min, the bacterial solution was centrifuged at 4°C and 12,000 rpm. The supernatant was used as an extracellular enzyme (EC) sample to determine activity. The precipitate was centrifuged and resuspended in 100 ml PBS buffer. An ultrasonic crusher was used to break up the bacteria cells in the suspension solution. The ultrasonic power was set at 300 w, repeated every 5 s, stood for 5 s, and lasted 5 min (JY98-IIIDN ultrasonic processor, Ningbo Xinzhi Biotechnology Co., Ltd.). The sample stranded for 5 min after the operation, then repeat the procedure. After ultrasonic treatment, the bacteria solution was centrifuged for 5 min at 4°C and 12,000 rpm. The supernatant was utilized to determine the activity of intracellular enzymes (IC).

##### 2.4.3.2. Determination of enzyme activity

###### 2.4.3.2.1. β-glucosidase

The enzyme activity was measured using Saha’s technique (Saha and Bothast, 1996). With a 50 mM pH 5.0 acetic acid buffer-β-D-galactopyranoside (p-npg) solution, 0.8 mM p-nitrophenyl was produced. A mixed solution of 0.1 ml of moderately diluted crude enzyme solution and 0.9 ml of 50 mM pH 5.0 acetic acid buffer solution was prepared. The mixed solution was preheated in 50°C constant temperature water bath for 5-10 min. Then, 1 ml of 0.8 mM p-nitrophenyl-β-D-galactopyranoside solution (P-NPG), preheated for 5–10 min, was added to the mixed solution. After 30 min of reaction, 1 ml 0.5 M of pre cooled Na_2_CO_3_ solution was added immediately to stop the reaction. The sample was standed for 5 min, and the optical absorption value (OD) at 405 nm was measured. The amount of enzyme in 1 ml Enzyme solution producing 1 uM p-nitrophenol in 1 min was defined as one unit of enzyme activity.

###### 2.4.3.2.2. Endoglucanase

The enzyme activity was measured using Haggag’s Method (Haggag, 2013) 0.1 ml of sodium carboxymethyl cellulose (1% w/V), 1 ml of 50 mm pH 7 phosphate citric acid buffer, and 1 ml of diluted crude enzyme solution were mixed. The reaction was conducted at 40°C for 30 min before being stopped with a 2 ml DNS solution. Instead of enzyme solution, phosphate citric acid buffer was introduced to the same system as blank solution. The reaction and blank solutions were boiled for 5 min at 100°C, then cooled, and 5 ml distilled water was added. The absorption value (OD) of the solution at 540 nm can be used to calculate the reducing sugar concentration. The endoglucanase activity of one unit (U) was defined as the amount of enzyme in 1 ml Enzyme solution that can degrade sodium cellulose and release 1 μMl glucose in 1 min.

###### 2.4.3.2.3. Exoglucanase

The enzyme activity was measured using Oliveira and Miller’s Method (Miller et al. 1959; Oliveira et al., 2013). 1 ml of 1% microcrystalline cellulose substrate solution, 1 ml of 100 mM pH5.0 citric acid buffer, and 1 ml of crude enzyme solution were mixed and reacted for 30 min at 50°C. The reaction was then stopped with 2 ml of DNS reagent. Instead of enzyme solution, the citric acid buffer was added to the same reaction system as the blank. The reaction and blank solutions were boiled for 5 min at 100°C, then cooled, and 5 ml distilled water was added. The absorption value (OD) of the solution at 540 nm can be used to calculate the reducing sugar concentration. The exoglucanase activity of one unit (U) was defined as the amount of enzyme in 1 mL Enzyme solution that can degrade sodium cellulose and release 1 μMl glucose in 1 min.

###### 2.4.3.2.4. Xylanase

The xylanase activity assay was based on the method described by Bailey et al. (1992). The reaction mixture，which consisted of 1.8 ml of a 1.0% (w/v) suspension of birch-wood xylan in 50 mmol L^−1^ sodium citrate at pH 5.3 and 0.2 ml of crude enzyme solution, was incubated for 5 min at 50°C. Released reducing sugar was determined by adding 3 ml of 3,5-dinitrosalicylic acid (DNS) solution and then incubated the mixture for 5 min at 95°C. Absorbance was measured at 540 nm. One unit (U) of xylanase was defined as the amount of enzyme required to catalyze the release of 0.01 μmol of xylose equivalent per minute.

###### 2.4.3.2.5. Lignin peroxidase

The activity of lignin peroxidase was determined by the method from Wan and Li (Caixia Wan, 2010). The volume of the total reaction system was 3 ml，which included 1.5 ml of 100 mM sodium tartrate buffer solution (pH = 4.5), 0.5 ml of 0.192 mM azure B, 0.5 ml of 0.6 mM H_2_O_2_ and 0.5 m of crude enzyme solution (final concentration of reaction system was 50 mM sodium tartrate buffer solution, 32 mM azure B, 0.1 mM H_2_O_2_).

The distilled water was used as blank solution, and the reaction solution containing inactivated crude enzyme after boiling was used as initial absorbance value, the change of absorbance value in the first 5 min was determined.

The formula of lignin peroxidase activity was as follows:


U/L=n×ΔOD/ε

[*n*: dilution times of enzyme solution; ΔOD: the change of absorbance value of reaction solution at 651 nm in each minute; ε: molar extinction coefficient (ε651 = 48,800 mol^−1^∙cm^−1^)].

###### 2.4.3.2.6. Manganese peroxidase

The enzyme activity was determined by guaiacol method from Hwang and Ann (2008). The volume of the total reaction system was 3 ml, which included 0.5 ml crude enzyme solution, 0.5 ml of 2.4 mM guaiacol, 0.5 ml of 1.2 mM manganese sulfate, 0.5 ml of 0.6 mM H_2_O_2_ and 1 ml of 150 mM sodium succinate buffer solution. The amount of enzyme causing UV absorption change at 465 nm in 1 min was one enzyme activity unit (U).

The formula of manganese peroxidase activity was as follows:


U/L=n×ΔOD/ε

[*n*: Dilution times of enzyme solution; ΔOD: the change of the absorbance value of the reaction solution at 465 nm per minute; ε: molar extinction coefficient (ε_465_ = 12,000 mol^−1^∙cm^−1^)].

###### 2.4.3.2.7. Laccase

The activity of laccase was determined by the method from Wan and Li (Caixia Wan, 2010). The volume of the total reaction system was 3 ml, which including 2 ml of 0.5 mM 2,2′-Azinobis [3-Ethylbenzthiazoline-6-Sulfonate (ABTS)], which was dissolved in 0.1 mM pH = 5.0 acetic acid sodium acetate buffer solution. The 1 ml of crude enzyme solution was added into the reaction system to start the reaction, and the absorbance value was measured at 420 nm. The amount of enzyme required to oxidize 1uM ABTS per minute was one enzyme activity unit (U).

The formula of laccase activity was as follows:


$$U/L=\left(\Lambda OD\times V1\times N\right)/\left(V2\times \Delta t\times \varepsilon \times 10^{-6}\right)$$

[ΔOD: Increment of absorbance at 420 nm; Δt: Variation of reaction time; V1: Total reaction volume (mL); V2: Volume of enzyme solution (mL); ε: molar extinction coefficient (ε_420_ = 36,000 mol^−1^∙cm^−1^); *n*: Dilution times of enzyme solution].

### 2.5. Effect of ZJW-6 on straw degradation

#### 2.5.1. Scanning electron microscope experiment

After 4 days of degradation, the straw material treated by the strain was removed, washed and filtered, and then dried at 60°C to constant weight in an oven. The dried straw samples that had been strained and the dried straw samples that had not been strained were crushed into powder with mortar and screened through a 40 mesh. The sample powder was fixed with 2% glutaraldehyde, rinsed with PBS buffer, dehydrated with an ethanol gradient, dried with a CO_2_ critical point dryer, and then loaded into an ion spatter for gold plating. After focusing, use scanning electron microscopy (SEM) to examine the sample’s surface properties. SEM images were captured with a 20 kV accelerating voltage and 1,000 times magnification. The microstructure of straw before and after treatment with ZJW-6 was observed and examined using the scanning electron microscope (SEM; Hitachi, s-4800, Japan).

#### 2.5.2. Fourier transform infrared spectroscopy analysis

The straw samples before and after treatment with ZJW-6 were dried in an oven at 105°C for 4 days before being ground to powder and stored in a desiccator for subsequent use. The average spectrum of the samples was obtained by scanning 50 times using a Fourier transform infrared spectrometer (Perkin Elmer, MA, United States) in the range of 400 cm^−1^ to 4,000 cm^−1^. Each sample had a spectral resolution of 2 cm^−1^.

### 2.6. Experiment on ZJW-6 promoting rice growth

The pot experiment was used to investigate the growth promoting effect of the ZJW-6 strain on rice plants. The physiological and biochemical indicators of paddy soil, rice roots, and plants during the seedling and tillering stages of rice were detected to assess the impacts of ZJW-6 strain on rice plants and rhizosphere soil.

After activation at LB medium, the seed solutions of ZJW-6 were centrifuged at 12,000 rpm for 5 min. The supernatant was extracted and centrifuged three times more. Bacteria were combined 1:2 with humic acid fertilizer, dried at room temperature, powdered, sealed, and stored at 4°C. Cylindrical opaque plastic buckets of 15 cm in diameter and 30 cm in height were selected and each bucket was filled with 5 kg of subsoil. Three separate replicates were set up: ZJW-6, humic acid application only (ck*), untreated (ck). The dried straw was cut into 10 cm sections and filled with 5 g each in nylon mesh bags, with 3 bags in each pot. Apply 8 g of mycorrhizal agent to the soil layer at a depth of 10-15 cm, (live bacteria number >1 billion pot^−1^). In each pot, make three holes (5 cm in diameter), place eight seeds in each hole, and then cover with a layer of fine soil after air-drying and sieving. After thorough watering, irrigation was performed every 2 days (three technical treatments × ten replications × two periods).

Rice soil, root, and above-ground associated indicators were measured and studied 30 and 60 days after mycorrhizal application. For soil indicators, the NaHCO_3_ leaching method was used to determine fast-acting phosphorus in the soil, the NaOH alkaline diffusion method was used to determine fast-acting nitrogen, and the potassium dichromate volumetric method-external heating method was used to determine organic matter in the soil. In the root index, root vigor was determined using triphenyltetrazolium chloride (TTC). In the above-ground part, the intact above-ground tissues and roots of rice were intercepted, washed with distilled water, and the surface water of the roots and above-ground parts blotted out using filter paper, dried, and weighed at 65°C to determine the root-shoot ratio (R/S). The aboveground net photosynthetic rate was measured with a portable photosynthesis measurement system (LI-6400XT, LI-COR, United States), and chlorophyll was measured with a chlorophyll analyzer (SPAD-502PLUS, Konica Minolta, Japan), which was used to record the chlorophyll content of the upper, middle, and lower parts of the leaves.

Rice varieties were obtained from Jilin Agricultural University Japonica conventional rice JINONG 667, Changchun (Jilin, China), humic acid fertilizer: humic acid ≥30%, organic matter ≥70%. n, P, K ≥ 5%, water solubility ≥60%, PH: 8 (Jiahe Co., Ltd., Heilongjiang, China).

## 3. Results

### 3.1. Screening and identification of *Cellulomonas* strain, ZJW-6

#### 3.1.1. Screening by straw degradation ability

**Figure 1:**
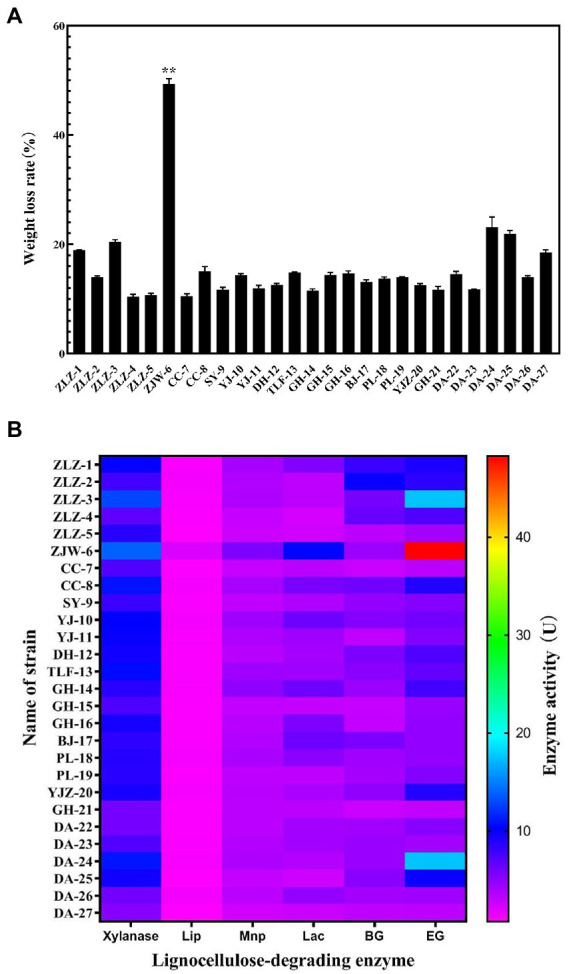
Characteristics of lignocellulose degradation of the strains isolated from soil samples. **(A)** The weight loss rate of rice straw after treated with 27 strains; **(B)** Heatmap of lignocellulose degrading enzyme activity of 27 strains.

Isolates from the original soil samples were inoculated on CMC-Na medium stained with Congo red dye, cellulose dissolving circles for each strain were obtained, according to their diameters, and 27 strains were selected and designated as shown in Figure 1. Figure 1 depicted the isolated strains’ ability to degrade rice straw. ZJW-6 was the most efficient in straw degradation among the 27 strains tested. In 4 days, the weight loss of rice straw was 49.3 and 49.6%, respectively. Meanwhile, as indicated in Figure 1B, the activities of the following six lignocellulose degrading enzymes were found in this study: xylanase, lignin peroxidase, manganese peroxidase, laccase β-Glucosidase, and endoglucanase. It is possible to conclude that the activity of strain ZJW-6’s xylanase, lignin peroxidase, manganese peroxidase, laccase, and endoglucanase was the highest among 27 strains, with values of 13.87 U/ml, 2.18 U/ml, 5.57 U/ml, 10 U/ml, and 48.27 U/ml, respectively. However, the β-glucosidase activity of ZJW-6 was relatively low. The properties of the ZJW-6 were explored further due to its exceptional performance.

#### 3.1.2. Optimization of straw degradation conditions of strain ZJW-6

**Figure 2:**
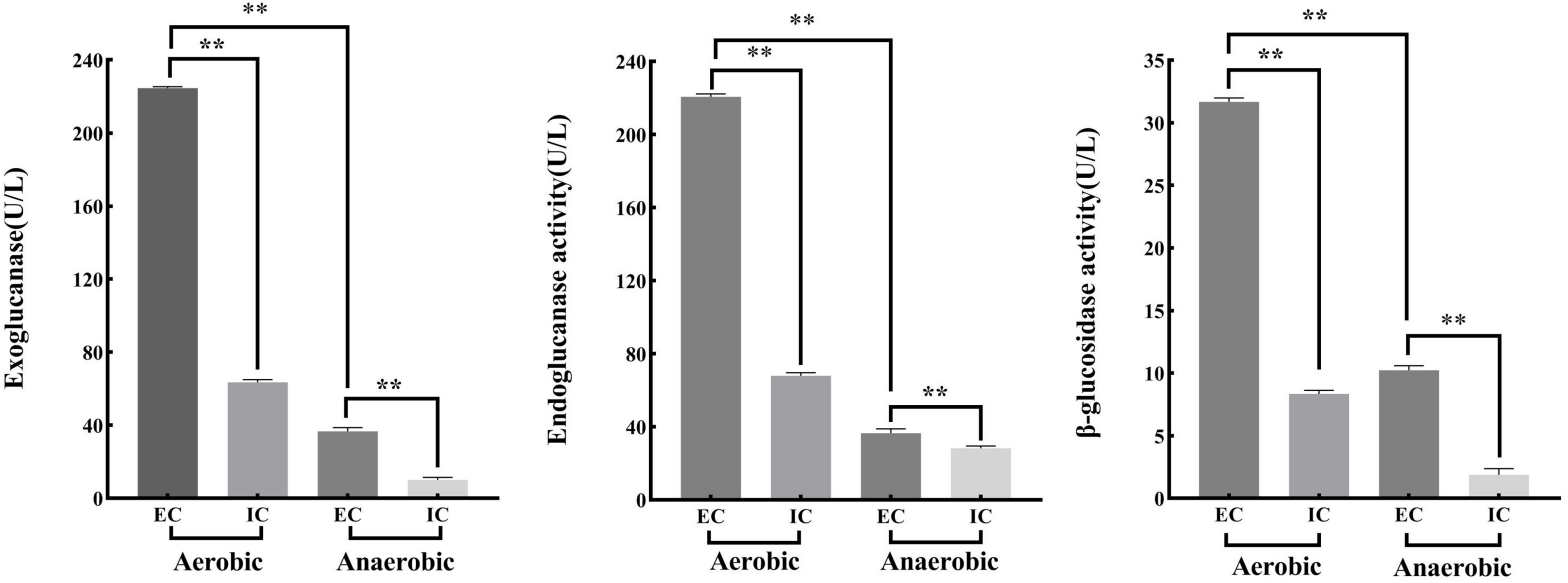
Optimal condtions of straw degradation of strain ZJW-6.

As indicated in Figure 2A, when nitrogen was used as the sole variable, strain ZJW-6 achieved the highest straw degradation rate of 52.6% when 8 g/L of nitrogen source was added to the culture; when pH was tested as a single component, the weight loss rate reached 52.5% (Figure 2B). When nitrogen was used as the sole variable in Figure 2C, strain ZJW-6 achieved the highest straw degradation rate of 52.6% when 8 g/L of nitrogen source was added to the culture; when temperature was the only variable in Figure 2C, the ZJW-6 bacterial culture displayed the maximum straw degradation efficiency at 31°C, with the strain having a weight loss rate of 51.93%.

**Figure 3:**
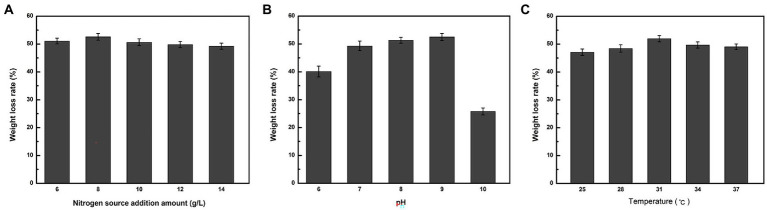
The response surface of the interaction of temperature, pH, and Nitrogen source addition amount of ZJW-6 on the degradation rate. **(A)** Response surface affected by Nitrogen source addition amount and pH interaction. **(B)** Response surface affected by Nitrogen source addition amount and temperature. **(C)** Response surface affected by pH and temperature.

According to the above single-factor test results, taking temperature (A), pH (B) and nitrogen source addition (C) as independent variables, the response surface was established by using design expert 8.0 software as shown in Figure 3. Based on the analysis of response surface, the optimal straw degradation conditions by ZJW-6 were determined as follows: nitrogen source addition, pH and temperature were 8.45 g/L, 8.57, and 31.63°C, respectively. Furthermore, the highest degradation rate could be 54.8%. Supplementary Table S2 depicts the results of the regression model’s variance analysis. The derived total regression model is highly significant (*p* < 0.01). In the model, factors B and C^2^ have a significant effect on the degradation rate (*p* < 0.01), and factors C and B^2^ have a significant effect on the degradation rate (*p* < 0.05). The fitting equation for the degradation rate of strain ZJW-6 was as follows:

$\begin{array}{l} \mathrm{Weight loss rate}\;\left(\%\right)=54.32-0.16A+1.63B+0.70C-0.11\mathrm{AB}\\ \;\;\;\;\;\;\;\;\;\;\;\;\;\;\;\;\;\;\;\;\;\;\;\;\;\;\;\;\;\;\;\;\;\;\;\;\;\;\;\;\;\;\;\;\;\;\;\;\;\;\;\;-0.45\mathrm{AC}-0.22\mathrm{BC}-0.42A^{2}-1.09B^{2}-1.66C^{2} \end{array}$

The optimal response surface conditions determined by software analysis were validated, and the degradation rate after three repeated experiments was 54.3%, which was close to the model’s predicted value. The weight loss rate was 4.7% higher than before optimization. The results demonstrated that the model could predict the ZJW-6 degradation of straw.

#### 3.1.3. Identification of the *Cellulomonas* strain, ZJW-6

**Figure 4:**
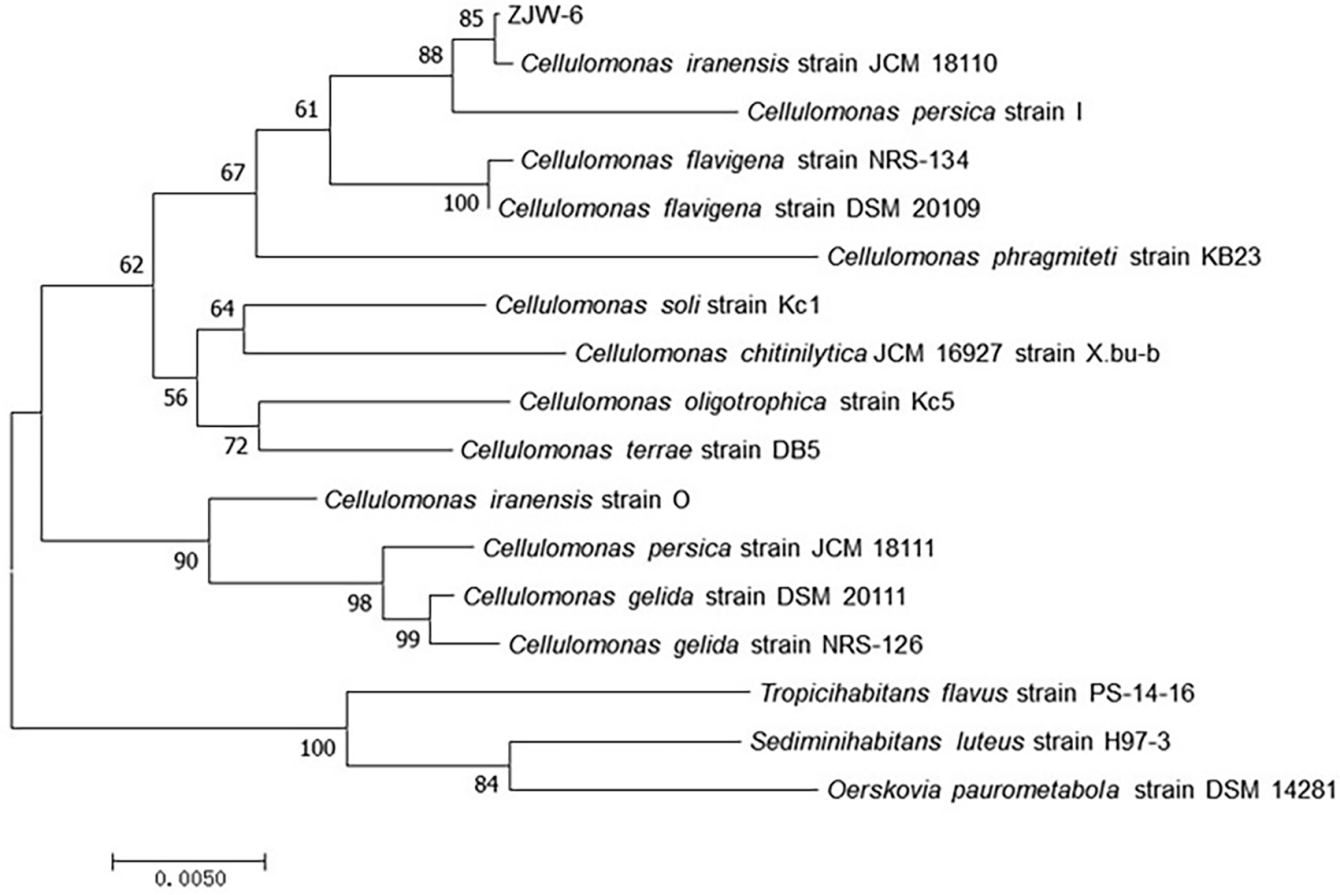
Neighborhood-joining tree based on 16S rRNA-encoding gene sequences of strain ZJW-6 and those species of *Cellulomonas* genus in NCBI database. The 16S rRNA-encoding gene sequences of *Tropicihabitans flavus*, *Sediminihabitans luteus* and *Oerskovia paurometabola* were used as outgroups. Only bootstrap values above 50% (percentages of 1,000 replications) are indicated. The numbers at nodes are bootstrap values from 1,000 replicates of the consensus tree. The scale bar represents 0.5% nucleotide sequence divergence. The GenBank accession numbers for the sequences used to generate the phylogenetic tree are as follows: *Cellulomonas iranensis* strain JCM 18110, NR 114319.1; *Cellulomonas persica* strain I, NR 024913.1; *Cellulomonas flavigena* strain NRS-134, NR 037027.1; *Cellulomonas flavigena* strain DSM 20109, NR 074490.1; *Cellulomonas phragmiteti* strain KB23, NR 108148.1; *Cellulomonas soli* strain Kc1, NR 113283.1; *Cellulomonas chitinilytica* JCM 16927 strain X.bu-b, NR 041511.1; *Cellulomonas oligotrophica* strain Kc5, NR 113284.1;*Cellulomonas terrae* strain DB5, NR 043257.1; *Cellulomonas iranensis* strain O, NR 024914.1; *Cellulomonas persica* strain JCM 18111, NR 114320.1; *Cellulomonas gelida* strain DSM 20111, NR 119159.2; *Cellulomonas gelida* strain NRS-126, NR 037026.1; *Tropicihabitans flavus* strain PS-14-16, NR 136455.1; *Sediminihabitans luteus* strain H97-3, NR 114322.1; *Oerskovia paurometabola* strain DSM 14281, NR 025471.1.

Pairwise alignments of the 16S rRNA-encoding gene sequence of ZJW-6 with the 16S rRNA-encoding gene sequences of type strain *Cellulomonas* species in the EMBL database were performed using the Genetics Computer Group’s Needleman/Wunsch alignment algorithm. ZJW-6 had the highest 99% similarity to the *Cellulomonas iranensis* sequence. The sequence similarity between strain ZJW-6 and other *Cellulomonas* species was investigated. The phylogenetic tree was constructed using the neighbor joining algorithm, and the tree’s robustness was determined using a bootstrap analysis that sampled the sequence 1,000 times. Outgroup controls included the of type strains of other species such as *Tropicihabitans flavus*, *Sediminihabitans luteus*, and *Oerskovia paurometabola*. The results demonstrated that strain ZJW-6 belonged to the *Cellulomonas* genus. Based on the results of the above study, strain ZJW-6 was identified as belonging to *Cellulomonas iranensis*. ZJW-6’s nucleotide sequence has been submitted to GenBank as accession number MW543304 (Figure 4).

The morphology of the bacterium ZJW-6 cultured on an LB solid plate was investigated, and Gram-staining was performed as described by Froböse et al. (2020). *Cellulomonas iranensis* ZJW-6 was Gram-positive and grew as a tiny, yellowish-green colony on agar, which was consistent with the *Cellulomonas iranensis*’ description by Elberson et al. (2000a).

### 3.2. The characteristics of ZJW-6

#### 3.2.1. Degradation of cellulose, hemicellulose and lignin of straw by strain ZJW-6

**Figure 5:**
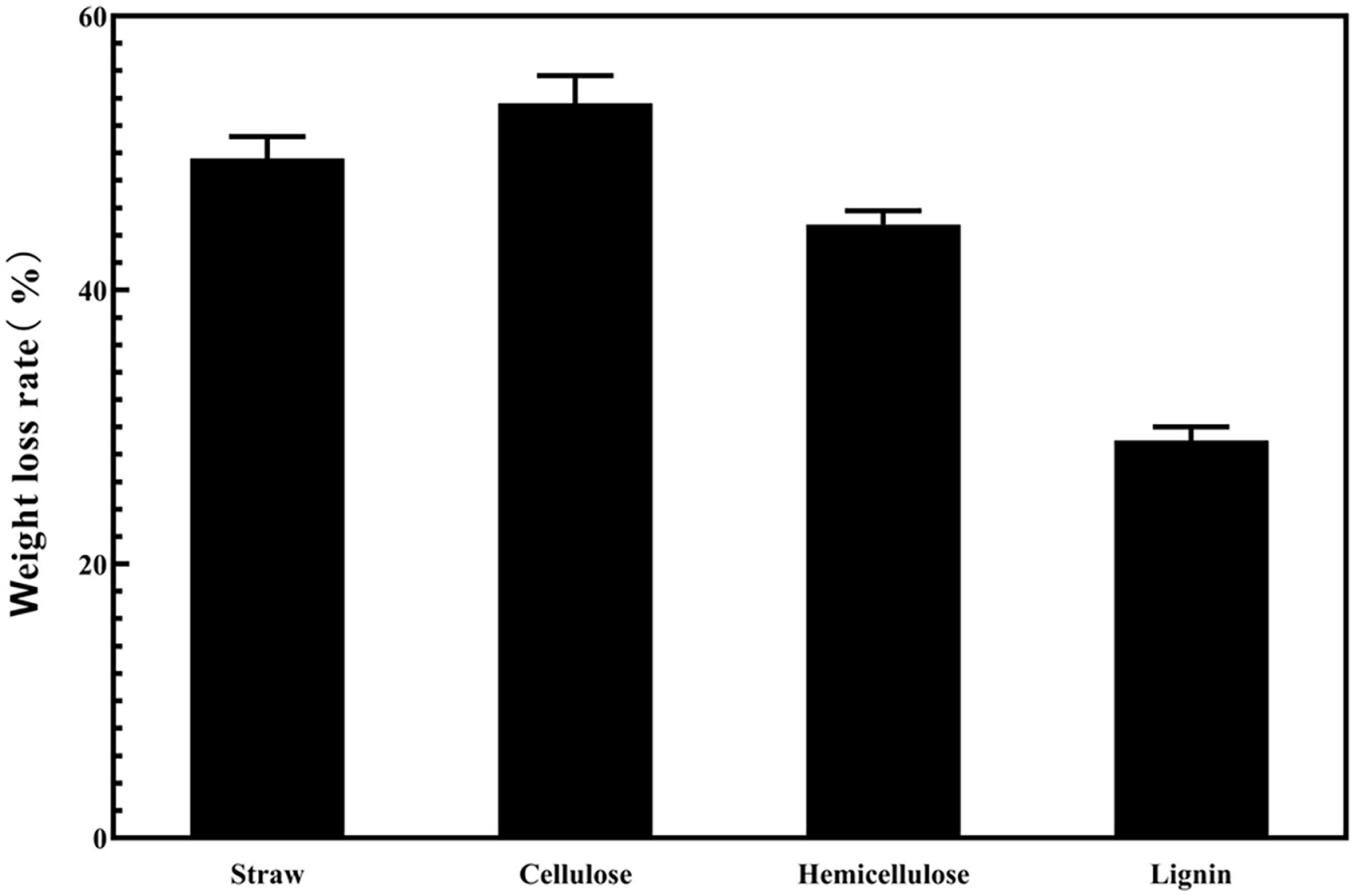
The weight loss rate of the different components in rice straw under the condition of ZJW-6.

Figure 5 shows that the strain was capable of degrading cellulose, hemicellulose, and lignin. The strain degraded rice straw over 4 days, and the weight loss rates of cellulose, hemicellulose and lignin were 43.6, 47.8, and 29%, respectively. Lignin had a significantly lower weight loss rate than cellulose and hemicellulose.

#### 3.2.2. Growth curves of ZJW-6 under aerobic and anaerobic conditions

Simultaneously, to elucidate the rationale for the strain’s change in cellulose degradation ability under aerobic and anaerobic circumstances, the strain’s growth under aerobic and anaerobic conditions was investigated.

**Figure 6:**
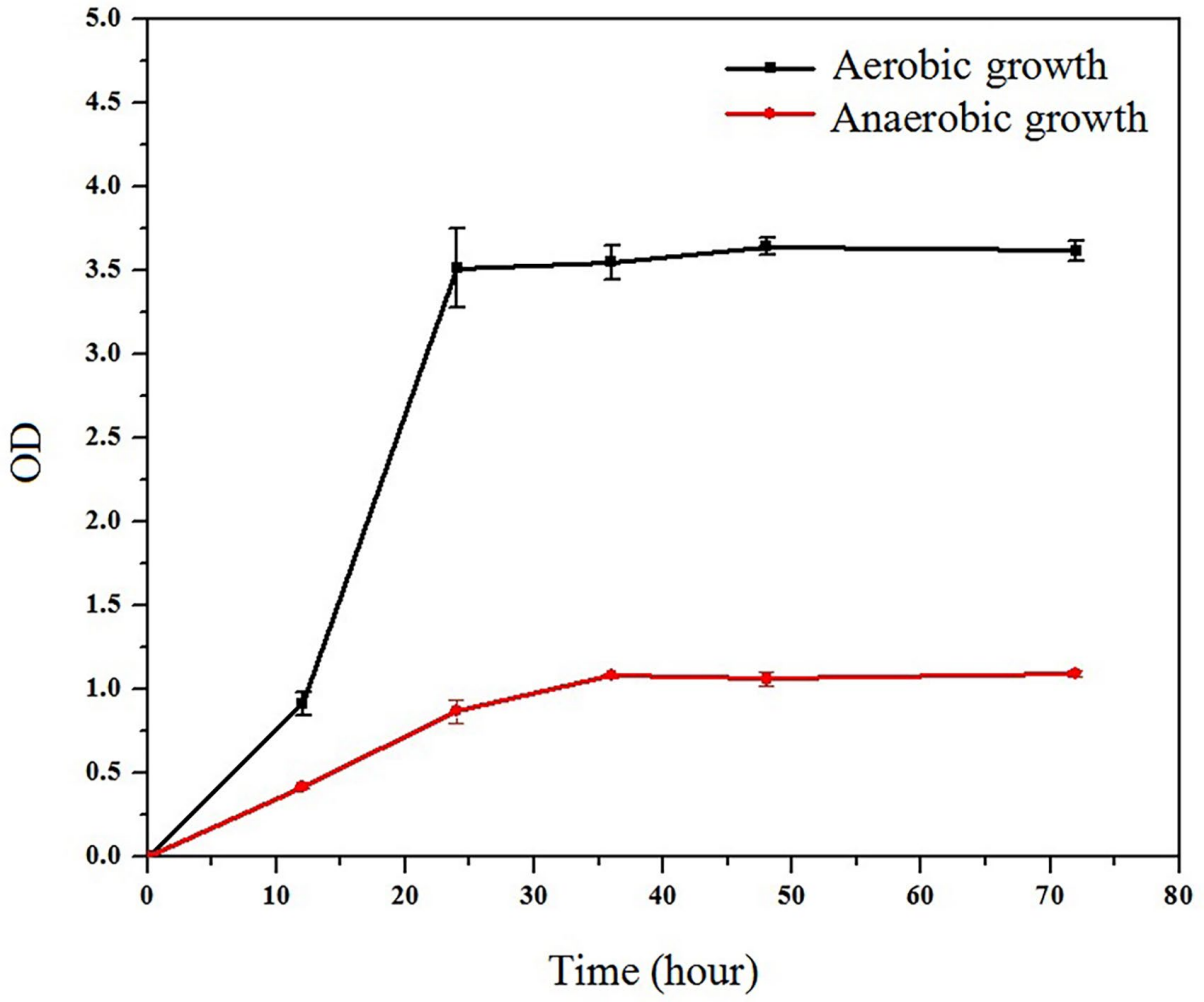
Growth curve of ZJW-6 under aerobic and anaerobic conditions.

The strain’s growth reached the plateau stage after 24 h under aerobic and anaerobic conditions, as illustrated in Figure 6. The strain grew significantly faster in aerobic conditions than in anaerobic conditions. However, when compared to most cellulose-degrading bacteria, the ZJW-6 strain has a good growth condition under anaerobic conditions, which is of great importance for the strain’s applicability in anaerobic fermentation processes.

#### 3.2.3. Cellulase characteristics of ZJW-6 under aerobic and anaerobic conditions

According to the above experiment findings, the strain ZJW-6 was identified as *Cellulomonas iranensis*. As Yuanyuan Tian et al. Reported in 2019, most *Cellulomonas* are cellulolytic and able to reduce nitrate. Furthermore, the function of microbial cellulose degradation is mostly dependent on exoglucanase, endoglucanase, and β-glucosidase (Yuanyuan Tian et al., 2019). The intracellular and extracellular activities of three cellulases from strain ZJW-6 were investigated in the following investigation under aerobic and anaerobic conditions.

**Figure 7:**
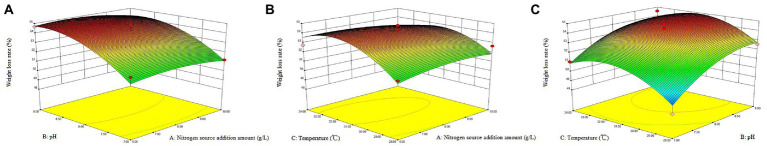
Cellulase activity of ZJW-6 under aerobic and anaerobic conditions. EC, extracellular enzymes; IC, intracellular enzymes. **(A)** Exoglucanase; **(B)** Endoglucanase; **(C)** β-glucosidase.

The following results can be obtained from Figure 7. The extracellular enzyme activities of exoglucanase, endoglucanase and β-glucosidase under aerobic conditions were 224.54 U/l, 220.68 U/l and 31.66 U/l, respectively. However, the extracellular enzyme activities of exoglucanase, endoglucanase and β-glucosidase under anaerobic conditions were 36.66 U/l, 36.56 U/l and 10.23 U/l. Meanwhile, the intracellular enzyme activities of exoglucanase, endoglucanase and β-glucosidase under aerobic conditions were 63.48 U/l, 67.94 U/l and 8.34 U/l, respectively. The intracellular enzyme activities of exoglucanase, endoglucanase and β-glucosidase under anaerobic conditions were 10.13 U/l, 28.34 U/l and 1.89 U/l.

Under aerobic conditions, the activities of cellulose degrading enzymes (including exoglucanase, endoglucanase and β-glucosidase) of strain ZJW-6 were significantly or extremely significantly greater than those under anaerobic conditions, whether intracellular or extracellular enzymes. Microbial anaerobic fermentation of straw can convert the lignocellulose in straw into nutrients for microbial growth, reducing the anaerobic fermentation period and improving the rate of straw fermentation and gas production. The strain ZJW-6 has great application value in the usage and development of straw biomass resources because it can produce cellulose degrading enzymes in both aerobic and anaerobic conditions (Tsapekos et al., 2017).

Moreover, glucosidase, endoglucanase, and exoglucanase extracellular enzyme activities were significantly or extremely significantly higher than intracellular enzyme activities. During the straw returning process, there is a significant correlation between the carbon and nitrogen content of soil and the activity of extracellular enzymes in soil. Strain ZJW-6 has excellent extracellular enzyme activity, indicating its potential application relevance in agricultural straw returning.

### 3.3. Effect of ZJW-6 on straw degradation

#### 3.3.1. Scanning electron microscope

**Figure 8:**
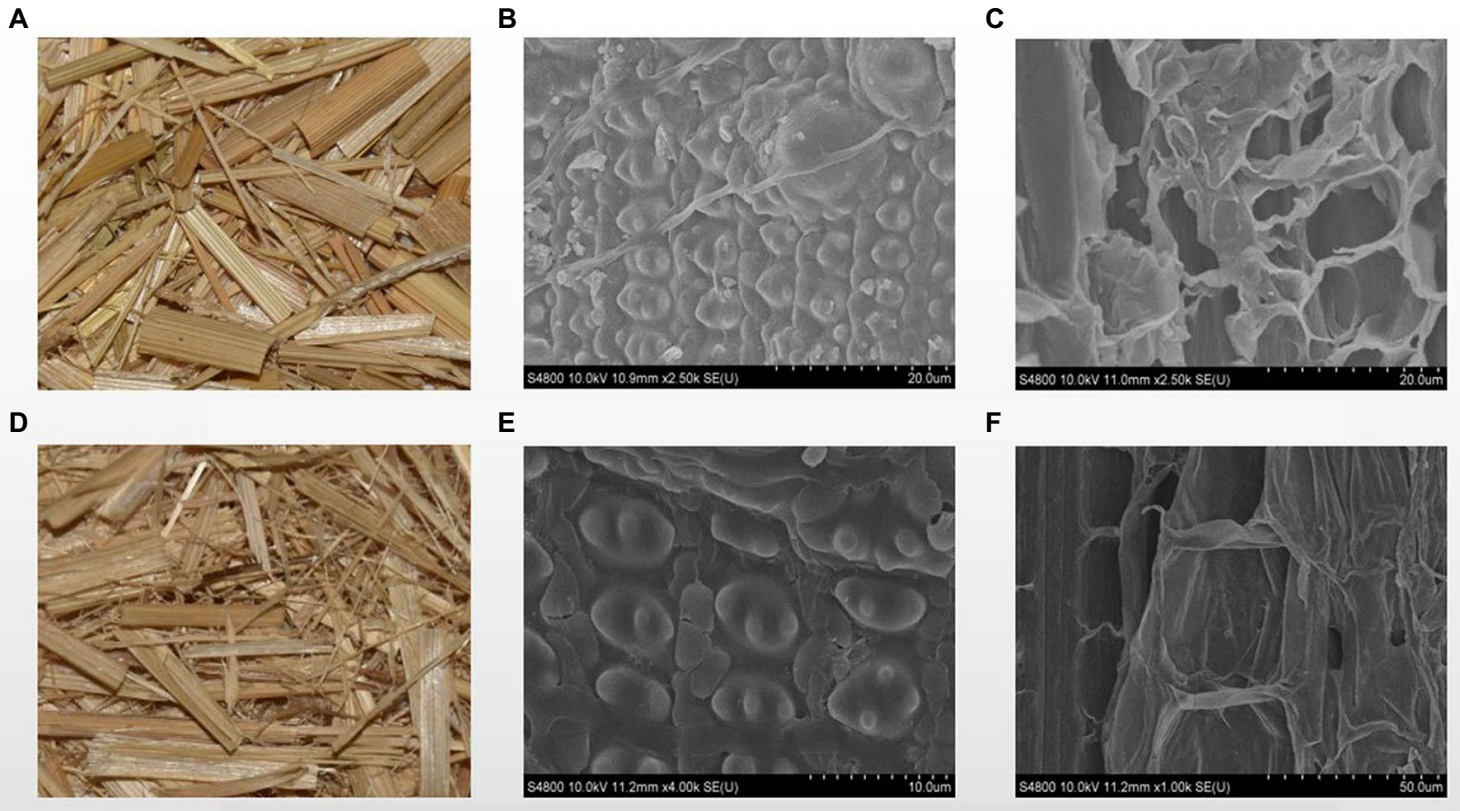
The SEM images of straw before and after treatment by ZJW-6. **(A)** straw before treatment; **(B)** the outer surface of straw before treatment; **(C)** the internal structure of straw before treatment; **(D)** straw after treatment; **(E)** the outer surface of straw after treatment; **(F)** internal structure of straw after treatment.

The exterior surface of rice straw was smooth before being treated with strain ZJW-6, while the inner surface was rough. The rice straw’s structure mostly consisted of epidermis, mechanical tissue, basic tissue, and a vascular bundle, as shown in Figures 8A–C. The primary component of rice straw was made up of parenchyma cells (Kaur et al., 2017). Figure 8C shows a loose wall with visible intercellular gap. Cellulose and hemicellulose were the primary components of the parenchyma cell wall (Kaur et al., 2017). The surface of straw showed mild damage after being treated by strain ZJW-6 due to the degradation of cellulose and hemicellulose, as shown in Figure 8E, the ordered straw structure was destroyed after the lignin connected cellulose and hemicellulose was decomposed. The highly compact microtubule bundle structure of straw loosened and was damaged to varied degrees. At the same time, the internal tissue also grew looser and thinner as illustrated in Figure 8F. The cellulose and hemicellulose loose structure was degraded, exposing the inner structure of straw.

#### 3.3.2. Fourier transform infrared spectroscopy

**Figure 9:**
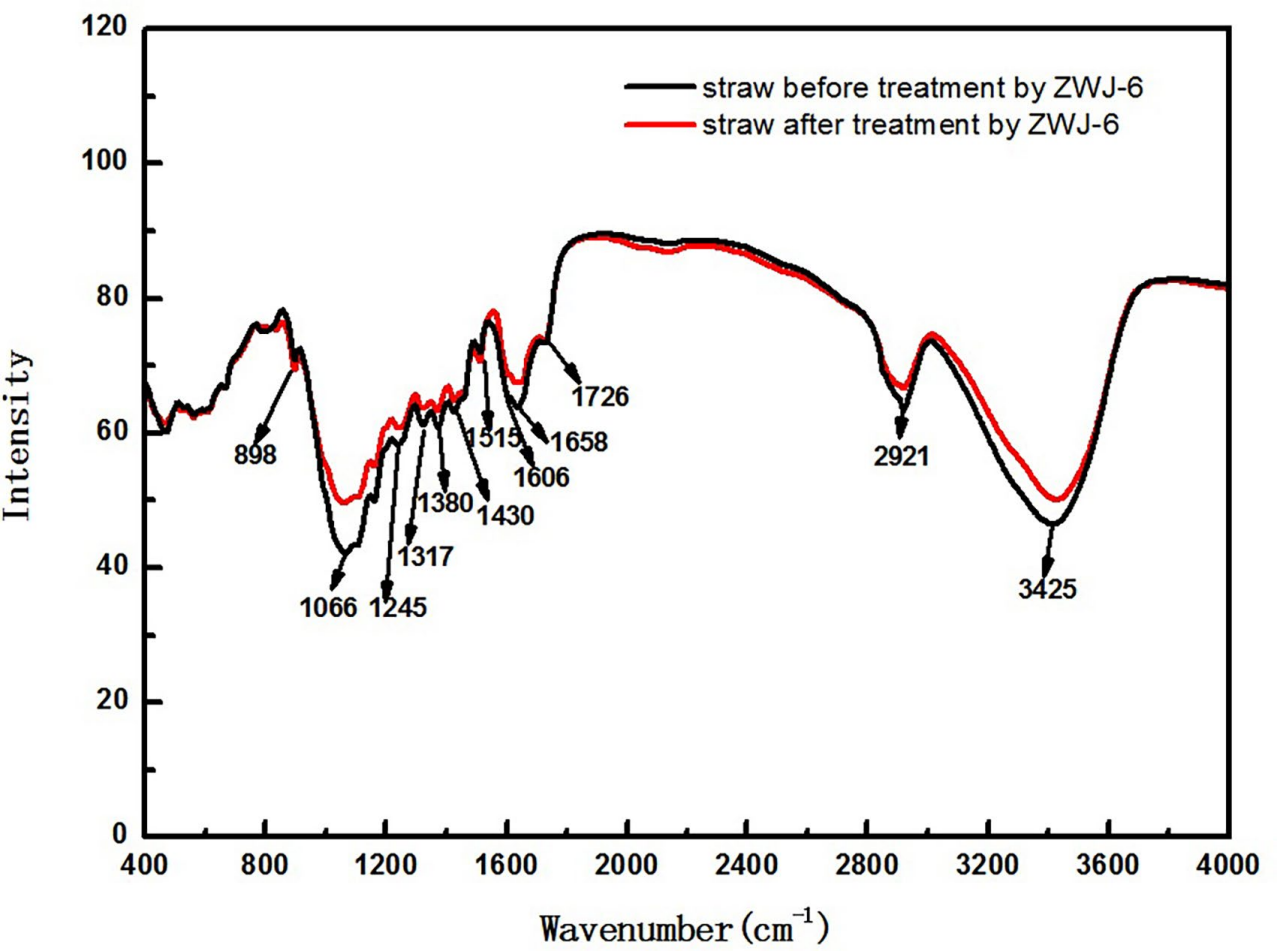
The infrared absorption spectra of straw before and after treated by ZJW-6.

The degradation process of crop straw is very complex, and the infrared spectrum has distinct properties that allow each compound’s infrared spectrum to be distinguished. From the infrared absorption spectra of straw samples in the range of 400 cm^−1^ to 4,000 cm^−1^, according to research by Xu Feng et al. (2007) and Kim et al. (2003), some inferences can be obtained, and the indications of infrared spectrum results are provided in the Table 1. By comparing the infrared absorption spectra of straw before and after ZJW-6 treatment in Figure 9, it is evident that the spectrum findings of straw have significant differences. The majority of the signals were weakened by straw degradation.

**Table 1:** Assignment of wavenumber value in the infrared absorption spectra of straw before and after treatment by ZJW-6.

Mode	Wavenumber (cm^−1^)	Assignment
1	898	Glycosidic bond stretch
2	1,066	C-0 stretching in hemicellulose and cellulose
3	1,245	The guaiacyl ring breathing with C–O stretching
4	1,317	Methoxyl C– H bending of lignin; syringyl and guaiacyl
5	1,380	Aliphatic C–H stretch in CH_3_ or CH_2_
6	1,430	Deformation of C-H bond plane caused by aromatic ring stretching
7	1,515	C-C stretching of aromatic rings
8	1,606	Stretching vibration of aromatic ring skeleton and C=N
9	1,658	The C-O stretch in conjugated ketone and carbonyl groups
10	1726	Acetyl group in hemicellulose structure and Phenyl ester bond between hemicellulose and lignin
11	2,921	Hydrogen bonds in cellulose and C-H bonds in methyl and methylene groups
12	3,425	O-H stretching in hemicellulose and cellulose

### 3.4. Growth promoting effect of ZJW-6 on rice

To investigate the potential of ZJW-6 combination in agriculture, ZJW-6 was used as bacterial fertilizer for rice pot experiments, and soil (fast-acting phosphorus, alkaline nitrogen, organic matter), root (root activity, root-shoot ratio) and aboveground (net photosynthetic rate, chlorophyll) indicators were assessed in two phases (seedling and tillering) of rice.

**Figure 10:**
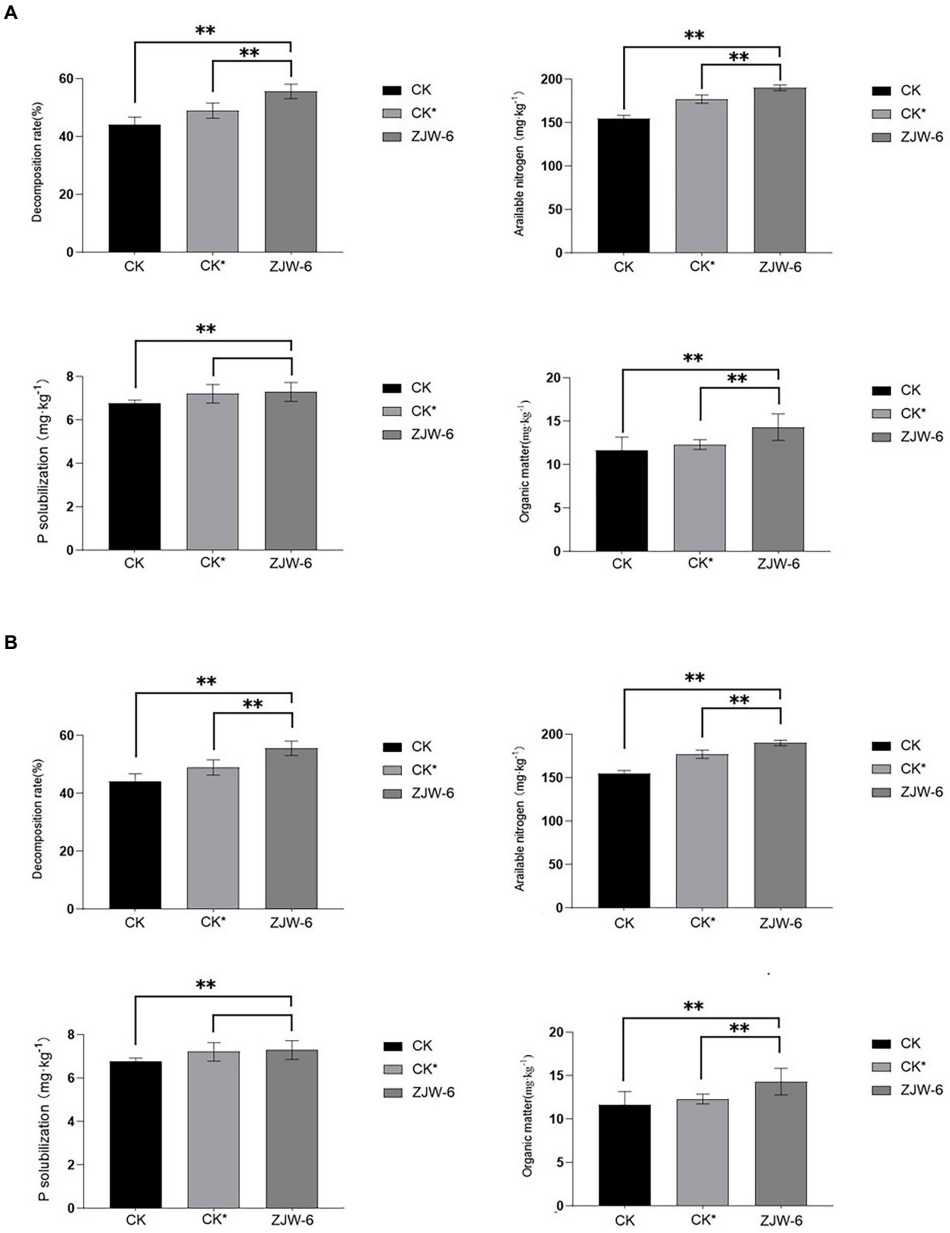
Index of soil quality. **(A)** seedling stage; **(B)** tillering stage. **p < 0.05.

Figure 10 depicts the organic matter contents of the soils of each treatment at the seedling and tillering stages. It was discovered that soil treated with ZJW-6 performed much better than soil treated with humic acid and soil treated without special treatment in terms of decomposition rate, alkali hydrolyzable nitrogen content, and organic matter content, regardless of seedling or tillering stage. Although there was no significant variation in available phosphorus content, ZJW-6 had the highest soil index.

**Figure 11:**
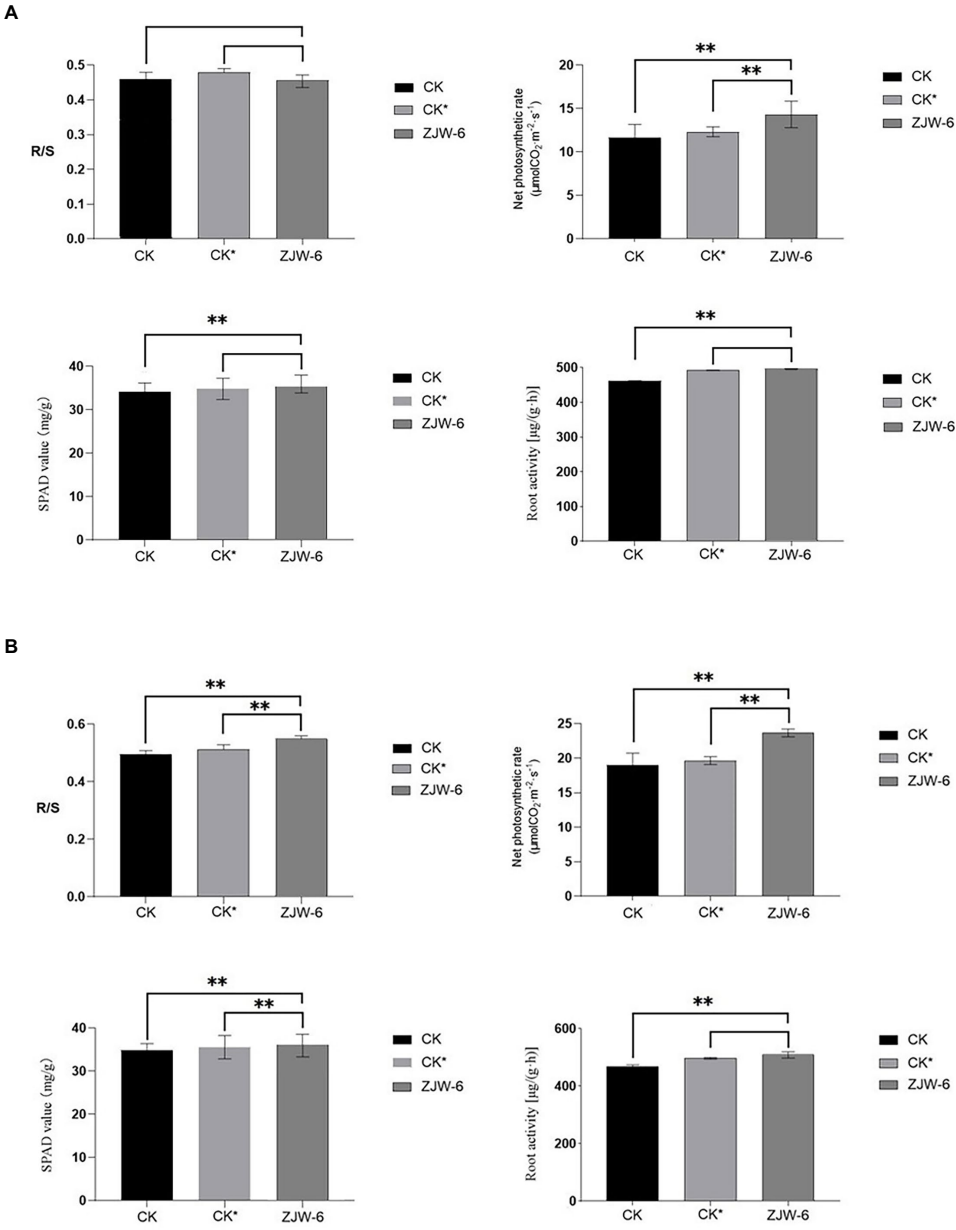
Physiological index of rice. **(A)** seedling stage; **(B)** tillering stage (R/S: root-shoot ratio). **p < 0.05.

The Figure 11 depicts the plant growth indicators. The net photosynthetic efficiency of plants treated with ZJW-6 is significantly higher than that of plants treated with humic acid and without special treatment, and the chlorophyll content and root activity indicators of plants treated with ZJW-6 are slightly higher but not significantly higher than those of plants treated with humic acid. Plants treated with ZJW-6 have much higher markers than plants that have not been treated. The root shoot ratio of plants treated with humic acid was the best at the seedling stage, while the root shoot ratio of plants treated with ZJW-6 was the highest at the tillering stage.

## 4. Discussion

In the natural environment, fungi are the microorganisms with the highest ability to degrade lignocellulose. Straw degrading fungi are classified as yeast or mold, with mold being the most studied, and including Penicillium, Aspergillus, Trichoderma, and white rot fungi. The degrading efficiency of typical fungi was around 40% after 12 days. Liu Qili and Xiaohui, for example, screened a cellulase fungus MC-1 from semi-rotten straw and semi-rotten wood. The weight loss rate after 45 days of straw treatment was 46.84% (Liu Qili and Xiaohui, 2014). Fungi development and application on a large scale are limited due to low efficiency, low enzyme activity, and pathogenicity (aspergillus) (Ahmad and Khare, 2018; Dollhofer et al., 2018; Jian Du et al., 2018). Bacteria have the advantages of a short propagation cycle, a simple structure, high stress resistance, and acid alkali resistance, all of which play essential roles in lignocellulose degradation (Rosnow et al., 2017). However, because of the complex and poor quality of farmland soil environment, most strains continue to experience some challenges, such as poor colonization ability, slow proliferation rate, instability and single effect, etc. More researchers are addressing these challenges by creating complex flora. Jiajin Liang, for example, isolated the microbial consortium oem2 from rice bran in 2018, which could degrade 41.5% of rice straw in 12 days (Liang et al., 2018). In 2005, Lu isolated a group of high temperature lignocellulose degrading bacteria tc-y from anaerobic digestion sludge and used it to degrade 49.5% corn straw in 20 days (Jun Lu et al., 2019). In a nutshell, it is still an important problem to screen degradation bacteria with better degradation efficiency and stronger environmental adaptability in the process of efficient crop straw utilization.

ZJW-6 performed excellently in the degradation of rice straw. In 4 days, weight loss of rice straw reached 49.3%. Compared with the typical lignocellulose degrading microorganisms, ZJW-6 has higher degradation ability. Its degradation capability is similar to that of a good lignocellulose degrading microbial community. Furthermore, if the environmental modification of straw degradation was appropriately regulated, the strain’s straw degradation ability might be further improved, potentially exceeding 54% in principle.

Based on the findings of the experiments, it was determined that ZJW-6 belongs to *Cellulomonas iranensis*. Bergey et al. described the *Cellulomonas* (Bergey et al., 1923), which was later revised by Clark and Stackebrandt et al. (Dworkin et al., 2006; Stackebrandt and Genus, 2012). This genus contains Gram-positive irregular rods with cellulolytic activity. They were cultured in aerobic conditions; however, most strains may also thrive in anaerobic environments (Chang-Muk Lee et al., 2008; Lagier et al., 2012). Most of them were originally isolated from environmental samples, and occasionally from rumen and activated sludge. Although the growth rate of ZJW-6 in aerobic environments was much higher than in anaerobic environments, the strain can grow well under anaerobic conditions, which is highly important for the strain’s applicability in anaerobic fermentation processes.

*Cellulomonas iranensis* was first isolated in 2000 from forest humus soil located under walnut, fifig, hornbeam, mimosa, and box trees in Iran’s Ramsar Forest (Elberson et al., 2000b). It has the ability to produce acid from dextrin but cannot use utilize ribose or raffinose. Moreover, it has a weak hydrolysis capability for starch and gelatin. To the best of our knowledge, *Cellulomonas iranensis* has not been determined to degrade straw well. To further investigate the process of ZJW-6 cellulose degradation, the activity properties of exoglucanase, endoglucanase, and-glucosidase secreted by ZJW-6 were investigated under aerobic and anaerobic environments. The main mechanism of cellulose degradation is the breakdown of chemical bonds by enzymes, resulting in cellulose structural cracking (Cragg et al., 2015; Quan Wang et al., 2017). Cellulases include exoglucanase, endoglucanase, and β-glucosidase. Endoglucanase cleaves cellulose chains internally in the amorphous regions. Exoglucanase produces cellobiose or glucose from the crystalline portion of cellulose. β-glucosidase hydrolyzes cellooligosaccharide to glucose. The exoglucanase and endoglucanase activity of ZJW-6 was the most prominent. This indicates that the strain has a high ability to degrade cellulose. Meanwhile, because rice straw has the largest cellulose concentration of any component, it has a direct impact on straw degradation.

After 4 days of treatment with the strain, the weight loss rates of cellulose, hemicellulose, and lignin in straw were 43.6, 47.8, and 29%, respectively. The results demonstrated that cellulose and hemicellulose degraded at a faster rate compared to lignin. Rice straw contains 36.5% cellulose, 27.7 percent hemicellulose, 12.3% lignin, and 13.3% ash (Hasunuma et al., 2013). Straw hemicellulose and cellulose are combined to produce a rigid cell network. Hemicellulose can only be thoroughly hydrolyzed after cellulose has been hydrolyzed (Bourquin et al., 2002). In general, bacteria that degrade cellulose may also degrade hemicellulose. Lignin provides structural support in the plant cell wall, while the intercellular matrix is filled with cellulose and hemicellulose to enhance the cell wall structure and resist microbial degradation. Straw’s intricate structure also demonstrates that lignin is the most difficult to degrade. Lignin’s degradation rate is usually lower than that of cellulose and hemicellulose.

It is necessary to observe the impact of straw degradation on the straw’s microstructure. Electron microscopy and Fourier transform infrared spectroscopy experiments were devised and carried out. Because of the degradation of cellulose and hemicellulose caused by ZJW-6 treatment, the surface of straw revealed a low degree of damage. Straw’s highly compact microtubule bundle structure loosened and was damaged to varied degrees. At the same time, the interior tissue began to loosen and thin. The changes in straw microstructure induced by straw structure degradation can be easily seen in electron microscope images. As indicated in Figures 8B,E, only a small amount of lignin in straw was degraded, and the difference was not statistically significant. However, due to the significant presence of noncrystalline cellulose and hemicellulose in the internal structure of straw, the degrading effect is visible and the change is relatively large. It can be seen by comparing the differences between Figures 8C,F.

According to the experimental results of infrared spectrum as displayed in Figure 9, the peak at 1658 cm^−1^ is the characteristic absorption peak of Lignocellulose. The relative absorption intensity of ZJW-6-treated straw was reduced in this band, indicating that some cellulose and hemicellulose were partially degraded. It is related to a decrease in lignocellulose content; the peak at 1380 cm^−1^ is the characteristic absorption peak of stretching vibration for - CH_3_ and - CH_2_, mostly due to some aliphatic and aliphatic compounds. With the degradation of straw by strain ZJW-6, the structure of aliphatic and aliphatic compounds was destroyed and decreased. As a result, the intensity of the absorption peak of straw treated with zwj-6 decreased at this peak, indicating that cellulose, hemicellulose, and lignin in straw were degraded to a lesser extent; the peak at 898 cm^−1^ is the characteristic peak of glycosidic bond, which is especially sensitive to changes in glycosidic bond in hemicellulose. The characteristic peak of ZJW-6-treated straw was clearly weakened, indicating that hemicellulose in ZJW-6-treated straw was clearly destroyed. The intensity of the peaks at 1245 cm^−1^, 1,317 cm^−1^, and 1,430 cm^−1^ decreased with the degradation of straw after treatment with zwj-6. These lignin-related bonds in straw were obviously degraded or even destroyed. It was discovered that the content of lignin varied throughout the degradation of straw. However, when compared to the lignin signal band, the peak intensity differences at 1066 cm^−1^, 1,606 cm^−1^, and 3,425 cm^−1^ are more noticeable. These peaks represent vibrations associated to cellulose and hemicellulose, indicating that the degradation degree of cellulose and hemicellulose is greater than that of lignin throughout the straw degradation process, which is also consistent with the strain’s enzymatic properties.

According to the findings of a rice pot experiment, the ZJW-6 strain could greatly increase rice soil quality during the seedling and tillering phases when straw was returned to the field. The content of organic matter, straw degraded rate, and alkali decomposed nitrogen increased by 22.9, 25.9, and 22.8%, respectively, during the seedling stage, while the content of available phosphorus increased by 7.5%. Compared to the corrosive acid treatment, the organic matter content, straw degradation rate, and alkali decomposed nitrogen rose by 16.2, 13.6, and 7.3%, respectively. The contents of organic matter, straw degradation rate, and alkali decomposed nitrogen increased by 22.8, 26.9, and 15.3%, respectively, at the tillering stage, while the content of available phosphorus increased by 7.0%. Compared with humic acid treatment, the contents of organic matter, straw decomposition rate, and alkali decomposed nitrogen rose by 17.33, 12.6 and 7.6%, respectively. Meanwhile, the ZJW-6 strain considerably promoted rice growth during the seedling and tillering stages. At the seedling and tillering stages, the net photosynthetic rate of rice plants treated with ZJW-6 increased by 22.9 and 24.6%, respectively, compared to no treatment and increased by 16.2 and 20.3%, respectively, compared to humic acid treatment; root activity increased by 7.5 and 4.6%, respectively, compared to no treatment and increased by 0.7 and 2.4%, respectively, compared to humic acid treatment. However, the chlorophyll concentration was marginally increased. The results showed that ZJW-6 accelerated the decomposition of straw and released the nutrient elements such as nitrogen, phosphorus and potassium into soil. As a result, the soil quality was improved, and the effective use of nutrients by plants was promoted, which promoted the growth of rice. It was determined that strain ZJW-6 could improve soil conditions and promote rice growth.

## Data availability statement

The datasets presented in this study can be found in online repositories. The names of the repository/repositories and accession number(s) can be found at: https://www.ncbi.nlm.nih.gov/, MW543304.

## Author contributions

XQ and YX completed the screening and identification of strains under the guidance of MY, LW, and XY. LW and SC were responsible for the research on the characteristics of strain ZJW-6 under the guidance of XY and MY. JS and YZ conducted a pot experiment on rice under the guidance of ZW, DW, and MW. LW and SC interpreted the data and wrote the manuscript with input from all co-authors. All authors contributed to the article and approved the submitted version.

## Funding

This study was supported and funded by the “Thirteenth Five-Year Plan” science and technology project of the Education Department of Jilin Province (JJKH20200335KJ) and the Science and Technology Development Plan Project of Jilin Province (20230508010RC).

## Conflict of interest

The authors declare that the research was conducted in the absence of any commercial or financial relationships that could be construed as a potential conflict of interest.

The reviewer ML declared a shared affiliation with the authors to the handling at the time of review.

## Publisher’s note

All claims expressed in this article are solely those of the authors and do not necessarily represent those of their affiliated organizations, or those of the publisher, the editors and the reviewers. Any product that may be evaluated in this article, or claim that may be made by its manufacturer, is not guaranteed or endorsed by the publisher.
