# Supplementary material for: Identification, characteristics and rice growth promotion of a highly efficient cellulolytic bacterial strain, Cellulomonas iranensis ZJW-6, isolated from paddy soil in central China
Source: Front Microbiol. 2023 Mar 22;14:1152966. doi: 10.3389/fmicb.2023.1152966 (PMC10073736; doi:10.3389/fmicb.2023.1152966)
Supplement: Supplementary file 1 [file Table_1.DOCX]

Supplementary Material

**Identification, characteristics and rice growth promotion of a highly efficient cellulolytic bacterial strain , *Cellulomonas iranensis* ZJW-6, isolated from paddy soil in central China**

**Lei Wu ^1^**^†^**，Songhao Che ^1^**^†^**, , Xueting Qin ^1^, Yufeng Xu ^1^, Shiqi Tian ^1^, Yuan Zhu ^1^, Jian Song ^1^, Dongchao Wang ^2^, Meikang Wu ^2^, Xue Yang ^1^, Zhihai Wu ^2^, Meiying Yang ^1*^**

*** Correspondence:** Meiying Yang [yangmeiying@jlau.edu.cn](mailto:yangmeiying@jlau.edu.cn)

**Material and Methods**

1. Gram stain experiment

The Gram staining experiment of ZJW-6 was carried out according to the following steps

(1) Smear: add a drop of sterilized normal saline to a clean slide, lift the colonies with the inoculation ring and smear them evenly.

(2) Drying and fixation: the coated side of the slide was upward, dried at room temperature, and fixed three times above the flame of alcohol lamp.

(3) Initial dyeing: add crystal violet dye on the bacterial membrane, and wash with water after 2 minutes.

(4) Mordant dyeing: wash with iodine solution and cover for about 60s.

(5) Decolorization: wash with 95% ethanol until the outflow ethanol is colorless, then wash with water immediately.

(6) Re staining: after 2 minutes of dripping and washing, it can be observed under microscope.

## Supplementary Table

Supplementary Table.1 Colour, morphological characteristics, source and Gram staining results of 27 strains

| mode | name | Colony colour | Colony morphology | Source of the strain | Gram staining  （Negative-/Positive+） |
| --- | --- | --- | --- | --- | --- |
| 1 | ZLZ-1 | Pale yellow | Round, edge translucent | Zhiluo Town, Shanxi Province  （E109.00，N36.00） | G^+^ |
| 2 | ZLZ-2 | White | Round, central divergence, transparent edge | Zhiluo Town, Shanxi Province  （E109.00，N36.00） | G^+^ |
| 3 | ZLZ-3 | Orange | Round, translucent | Zhiluo Town, Shanxi Province  （E109.00，N36.00） | G^+^ |
| 4 | ZLZ-4 | Bright yellow | Round, opaque | Zhiluo Town, Shanxi Province  （E109.00，N36.00） | G^+^ |
| 5 | ZLZ-5 | White | Small,irregular shape, opaque | Zhiluo Town, Shanxi Province  （E109.00，N36.00） | G^+^ |
| 6 | ZJW-6 | Chartreuse | Small, Round, translucent | Zhangjiawan, Shaanxi Province（E109.90，N36.61） | G^+^ |
| 7 | CC-7 | Creamy white | Round | Nanguan District, Changchun City, Jilin Province  （E125.35，N43.86） | G^+^ |
| 8 | CC-8 | Chartreuse | Round, translucent | Nanguan District, Changchun City, Jilin Province  （E125.35，N43.86） | G^+^ |
| 9 | SY-9 | Chartreuse | Round, translucent | Shuangyang District, Changchun City, Jilin Province  （E125.66，N43.53） | G^+^ |
| 10 | YJ-10 | Chartreuse | Small, irregular shape, translucent | Yanji City, Jilin Province  （E129.51，N42.89） | G^-^ |
| 11 | YJ-11 | Creamy white | irregular shape, Edge translucent | Yanji City, Jilin Province  （E129.51，N42.89） | G^+^ |
| 12 | DH-12 | Creamy white | Small, irregular shape | Dehui City, Jilin Province（E125.73，N44.52） | G^+^ |
| 13 | TLF-13 | Pale yellow | irregular shape | Turufan City, Xinjiang Uygur  Autonomous region  （E89.19，N42.95） | G^+^ |
| 14 | GH-14 | Pale yellow | Small, irregular shape, translucent | Genhe City,  the Inner Mongolia   Autonomous region  （E121.52，N50.78） | G^+^ |
| 15 | GH-15 | Creamy white | Small, irregular shape | Genhe City,  the Inner Mongolia   Autonomous region  （E121.52，N50.78） | G^+^ |
| 16 | GH-16 | Pale yellow | Small, irregular shape, translucent | Genhe City,  the Inner Mongolia   Autonomous region  （E121.52，N50.78） | G^+^ |
| 17 | BJ-17 | White | irregular shape | Xicheng District, Beijing City （E116.37，N39.91） | G^+^ |
| 18 | PL-18 | Pale yellow | irregular shape, translucent | Pingliang City, Gansu Province（E106.68，N35.54） | G^+^ |
| 19 | PL-19 | Creamy white | irregular shape | Pingliang City, Gansu Province（E106.68，N35.54） | G^-^ |
| 20 | YJZ-20 | Lavender | Round, edge translucent | Yangji Town, Shandong Province  (E106.68，N35.54) | G^+^ |
| 21 | GH-21 | Beige | Small, round | Qinhuangdao City, Hebei Province（E119.48，N39.84） | G^+^ |
| 22 | DA-22 | Beige | irregular shape | Da'an County, Baicheng City,  Jilin Province  (E124.29，N45.51) | G^+^ |
| 23 | DA-23 | White | round | Da'an County, Baicheng City,  Jilin Province  (E124.29，N45.51) | G^+^ |
| 24 | DA-24 | Orange | Round, edge translucent | Da'an County, Baicheng City,  Jilin Province  (E124.29，N45.51) | G^+^ |
| 25 | DA-25 | White | Round, edge translucent | Da'an County, Baicheng City,  Jilin Province  (E124.29，N45.51) | G^+^ |
| 26 | DA-26 | Creamy white | Round, transparent | Da'an County, Baicheng City,  Jilin Province  (E124.29，N45.51) | G^+^ |
| 27 | DA-27 | Creamy white | Round, transparent | Da'an County, Baicheng City,  Jilin Province  (E124.29，N45.51) | G^+^ |

Supplementary Table.2 Variance analysis of response surface regression model of strain ZJW-6

| Source | Sum of squares | degree of freedom | Mean square error | F-value | P-value | Significant |
| --- | --- | --- | --- | --- | --- | --- |
| Model | 45.21 | 9 | 5.02 | 8.02 | 0.0056 | ** |
| A | 0.20 | 1 | 0.20 | 0.32 | 0.5899 |  |
| B | 21.29 | 1 | 21.29 | 34.77 | 0.0006 | ** |
| C | 3.92 | 1 | 3.92 | 6.40 | 0.0392 | * |
| AB | 0.051 | 1 | 0.051 | 0.083 | 0.7820 |  |
| AC | 0.81 | 1 | 0.81 | 1.32 | 0.2878 |  |
| BC | 0.20 | 1 | 0.20 | 0.33 | 0.5832 |  |
| A^2^ | 0.73 | 1 | 0.73 | 1.19 | 0.3112 |  |
| B^2^ | 5.01 | 1 | 5.01 | 8.19 | 0.0243 | * |
| C^2^ | 11.52 | 1 | 11.52 | 18.81 | 0.0034 | ** |
| Residual | 4.29 | 7 | 0.61 |  |  |  |
| Loss of fit | 3.78 | 3 | 1.26 | 10.03 | 0.0248 | * |
| Pure error | 0.50 | 4 | 0.13 |  |  |  |
| Total | 49.50 | 16 |  |  |  |  |

Note: **: P<0.01; *: P<0.05.
